# Psychological Changes following Weight Loss in Overweight and Obese Adults: A Prospective Cohort Study

**DOI:** 10.1371/journal.pone.0104552

**Published:** 2014-08-06

**Authors:** Sarah E. Jackson, Andrew Steptoe, Rebecca J. Beeken, Mika Kivimaki, Jane Wardle

**Affiliations:** Department of Epidemiology and Public Health, University College London, London, United Kingdom; Erasmus University Rotterdam, Netherlands

## Abstract

**Background:**

Participation in weight loss programs is often associated with improved wellbeing alongside reduced cardio-metabolic risk. In contrast, population-based analyses have found no evidence of psychological benefits of weight loss, but this may be due to inclusion of healthy-weight individuals. We therefore examined cardio-metabolic and psychological changes following weight loss in a cohort of overweight/obese adults.

**Methods:**

Data were from 1,979 overweight and obese adults (BMI ≥25 kg/m^2^; age ≥50 y), free of long-standing illness or clinical depression at baseline, from the English Longitudinal Study of Ageing. Participants were grouped according to four-year weight change into those losing ≥5% weight, those gaining ≥5%, and those whose weight was stable within 5%. Logistic regression examined changes in depressed mood (eight-item Center for Epidemiologic Studies Depression score ≥4), low wellbeing (Satisfaction With Life Scale score <20), hypertension (systolic blood pressure ≥140 mmHg or anti-hypertensives), and high triglycerides (≥1.7 mmol/l), controlling for demographic variables, weight loss intention, and baseline characteristics.

**Results:**

The proportion of participants with depressed mood increased more in the weight loss than weight stable or weight gain groups (+289%, +86%, +62% respectively; odds ratio [OR] for weight loss vs. weight stable = 1.78 [95% CI 1.29–2.47]). The proportion with low wellbeing also increased more in the weight loss group (+31%, +22%, −4%), but the difference was not statistically significant (OR = 1.16 [0.81–1.66]). Hypertension and high triglyceride prevalence decreased in weight losers and increased in weight gainers (−28%, 4%, +18%; OR = 0.61 [0.45–0.83]; −47%, −13%, +5%; OR = 0.41 [0.28–0.60]). All effects persisted in analyses adjusting for illness and life stress during the weight loss period.

**Conclusions:**

Weight loss over four years in initially healthy overweight/obese older adults was associated with reduction in cardio-metabolic risk but no psychological benefit, even when changes in health and life stresses were accounted for. These results highlight the need to investigate the emotional consequences of weight loss.

## Background

The rising prevalence of weight-related diseases has led health organizations worldwide to advise overweight and obese adults to reduce their body weight [1], [2]. Recent evidence suggests that the majority are making attempts to lose weight. In a US survey, 60% of obese adults reported having tried to lose weight in the previous year [3], and a UK survey found that 60% of overweight and obese adults were ‘trying to lose weight’ [4]. Investigations of the biological and psychological effects of weight loss in the population context are therefore important for understanding costs and benefits.

Data from population-based cohort studies demonstrate that overweight and obese adults who lose weight benefit from improvements in cardio-metabolic risk [5], [6] similar to the effects reported in weight loss trials [7]–[9]. Recent events in Cuba illustrate the dramatic reductions in diabetes incidence and mortality during a period of economic crisis that produced an average 5.5 kg weight loss over the whole population; which reversed just as dramatically when economic conditions improved and population weights rebounded [10].

Effects on psychological wellbeing are less straightforward. In the clinical trial context, weight loss treatment is typically associated with a modest reduction in depression [11], [12]; although the extent of the reduction is not correlated with weight loss [12]. However, in two population-based cohort studies there was no evidence for beneficial psychological effects of weight loss; rather, evidence for unfavorable effects of weight loss was reported. In the Health and Retirement Study, weight loss over two years was associated with no change in depression in women and a slight *increase* in depressive symptoms in men [13]. In the Health ABC study, weight loss over three years was associated with increased odds of depressed mood; an effect that was maintained after controlling for weight loss intention [14]. However, both these studies included healthy weight as well as overweight and obese individuals, and it is possible that the adverse psychological effects were driven largely by effects in healthy weight participants for whom weight loss would not be recommended. In addition, while both studies adjusted for the number of chronic diseases, neither adjusted for potential confounding by the *onset* of illness over the weight loss period, and no attempt was made to address the possibility that weight loss and worsening mood might have been caused by other non-health-related factors, such as life stress.

The aim of this study was therefore to examine associations between changes in weight and changes in mood and wellbeing in a population sample of overweight and obese adults free from depression and serious illness at baseline. Cardio-metabolic changes were also examined to determine that participants were deriving typical benefits from weight loss and to facilitate comparisons with the trial literature. All analyses were repeated adjusting for changes in health status and major life events that occurred over the weight loss period in order to test for confounding.

## Methods

### Study population

Data for these analyses are from overweight and obese participants in the English Longitudinal Study of Ageing (ELSA), a population-based study of UK adults aged 50 or older [15]. The initial ELSA sample was drawn from households with one or more member age ≥50 years responding to the Health Survey for England (HSE) in 1998, 1999, and 2001. ELSA participants take part in biennial assessment waves, with objective measures of health status taken in alternate waves (every four years). Waves 2 and 4 (2004/5 and 2008/9) were the first two waves to include a nurse visit to take anthropometric measures and assess markers of health risk. We therefore examined associations with weight change over this four-year interval, with wave 2 constituting the baseline and wave 4 constituting the follow-up for the present analyses. Reliable weight measurements at both times were available for 4739 participants, of whom 2858 (60%) were overweight or obese (BMI ≥25 kg/m^2^) at baseline. Respondents reporting a diagnosis of clinical depression (*n* = 60) or a limiting long-standing illness (*n* = 857) at baseline were excluded to avoid confounding, leaving a final sample for analysis of 1979 men and women.

ELSA has received approval from various ethics committees, including the London Multi-Centre Research Ethics Committee. Full informed consent has been obtained from all participants. Data are publicly available at http://discover.ukdataservice.ac.uk.

### Measures

#### Anthropometric measurements

At baseline and follow-up visits, nurses measured height to the nearest millimeter using a portable stadiometer and weight to the nearest 0.1 kg using Tanita THD-305 portable electronic scales. Waist circumference was measured to the nearest even millimeter. Nurses also recorded any factors that could compromise measurement reliability (e.g. participant was stooped or unwilling to remove shoes). Anthropometric data judged by the nurse to be unreliable were excluded. In addition, we excluded cases with a weight change ≥10 kg if the waist circumference change was not consistent (*n* = 65).

#### Psychological variables

Depressed mood was assessed with an eight-item version of the Center for Epidemiologic Studies Depression Scale (CES-D) [16]. This asks about feelings over the last week (e.g. *“Over the last week have you felt sad”*), with binary response options (yes/no). Positively-framed items were reverse scored and all items summed to create a total score of 0–8, with higher scores indicating a greater number of depressive symptoms. The eight-item version has comparable validity and reliability to the original 20-item CES-D [17]–[19]. A CES-D score of 4 or higher is an established indicative threshold for presence of depressed mood [18].

As a secondary psychological outcome, we indexed wellbeing with the Satisfaction With Life Scale (SWLS) [20]. This asks respondents to indicate the extent to which they agree with five statements: *“In most ways my life is close to my ideal”*; *“The conditions of my life are excellent”*; *“I am satisfied with my life”*; *“So far I have got the important things I want in life”*; *“If I could live my life again, I would change almost nothing”*. Responses were on a Likert scale from 1 (strongly disagree) to 7 (strongly agree), and summed to produce a total score (range: 5–35) [21]. In the absence of an established cut-off, we used a score of below 20 to indicate low wellbeing because, like the CES-D threshold used to indicate depressed mood, it distinguishes between scores in the upper and lower half of the scale. Additional analyses were carried out using continuous scores to ensure that this effect was not due to the choice of cut-off.

#### Cardio-metabolic indicators

Two cardio-metabolic indicators were selected for inclusion in this analysis: systolic blood pressure and serum triglyceride level. Systolic blood pressure was chosen because it is one of the best established risk factors both for coronary heart disease and stroke across the life course [22], [23]. Similarly, serum triglyceride concentration is a consistent risk factor for cardiovascular disease [24]. Additionally, Mendelian randomization studies indicate that triglycerides play a causal role in coronary heart disease, rather than merely being correlates of risk [25]. Both fasting and non-fasting triglycerides predict risk [26], which is an advantage in a study like ELSA where fasting status is not established objectively. Analyses were also run using other cardio-metabolic indicators (diastolic blood pressure, total cholesterol, and LDL cholesterol) to check that effects were not unique to the variables selected for inclusion in this study to represent cardio-metabolic risk, and the results were very similar for these parameters.

Systolic blood pressure was measured using the Omron HEM-907 blood pressure monitor. After participants were seated for at least five minutes, three readings were taken at one-minute intervals and the mean of the second and third used. Fasting blood samples were taken from all consenting participants (83% at baseline and 77% at follow-up) for analysis of serum triglycerides. Detailed information on the analyses and quality control protocols are provided in the 2004 HSE technical report [27]. Baseline and follow-up samples were analyzed by the same laboratory following the same guidelines and protocols. We used established cut-offs to indicate hypertension and high serum triglycerides: systolic blood pressure ≥140 mmHg [28] and serum triglycerides ≥1.7 mmol/l [29]. As is conventional, individuals who reported being prescribed anti-hypertensive drugs were also included in the hypertensive category.

#### Demographic variables

Age and sex were included as control variables, as was household non-pension wealth which was used as an indicator of socioeconomic status because it has been identified as being a particularly sensitive indicator in this age group [30].

#### Weight loss intention

Intention to lose weight was also included as a control variable. This was assessed with the question: “*At the present time are you trying to lose weight, trying to gain weight, or are you not trying to change your weight*”, measured in the 1998 HSE, from which around a third of the original ELSA sample was recruited. Data were dichotomized to distinguish between participants who had been trying (intended) to lose weight and those who had not. Data were missing for participants drawn from 1999 and 2001 HSE (*n* = 1179, 60% of the sample) as this question was not included, so missing values were imputed. A multiple imputation model was run with age, sex, and weight status (all measured at wave 0; the wave of HSE from which participants were recruited into ELSA) as predictors, and weight loss intention as the imputed variable. The Hosmer-Lemeshow test for goodness of fit was non-significant (*p* = .985), indicating that the predicted and observed values did not differ significantly and the imputation model provided an adequate fit for the data. Five imputed data sets were created, each was analyzed separately, and the results were combined to produce pooled estimates of effects; allowing the analyses to account for uncertainty caused by estimating missing data. The pooled estimates were used in the analyses reported in this paper.

#### Health conditions

Limiting long-standing illness was assessed at baseline and follow-up with two questions: 1) *“Do you have any long-standing illness, disability, or infirmity? By long-standing I mean anything that has troubled you over a period of time or that is likely to affect you over a period of time”*; if they responded “yes” they were asked: 2) *“Does this illness or disability limit your activities in any way”*. Affirmation of a long-standing illness and any form of limitation classified the participant as having a limiting long-standing illness. Those who had a limiting long-standing illness at baseline were excluded from the current sample.

Whether participants had ever received a diagnosis of clinical depression was assessed by presenting a list of conditions (including depression) and asking: “*Has a doctor ever told you that you have (or have had) any of the conditions on this card*”. Participants answering “yes” to depression at baseline were excluded from the current sample. Participants also reported whether they had (or had ever had) doctor-diagnosed diabetes, coronary heart disease, cancer/malignant tumour (excluding minor skin cancers), chronic lung disease, and stroke, in response to the same question at each time point.

Mortality data were available through March 2011 (21 months after the follow-up examination) from the National Health Service central data registry, providing insight into potential undiagnosed disease.

#### Major life events

Inventories of major life events identify a large and varied number of events thought to be stressful that might therefore have an impact on weight and wellbeing, such as the death of a spouse, divorce, pregnancy, change in employment, and legal troubles [31]. The data available in ELSA made it possible to quantify the occurrence of three important major life events between baseline and follow-up: marital breakdown, being widowed, and the death of one or both parents. Marital breakdown was defined as being married at baseline and either divorced or legally separated at follow-up. Likewise, being widowed was defined as being married at baseline and widowed at follow-up. The death of a parent was indicated by responding “yes” to the question “*Is your natural mother/father still alive*” at baseline and responding “no” at follow-up. Due to low numbers, data on death of a mother or father were combined to reflect the death of one or both parents between baseline and follow-up.

### Statistical analysis

Respondents were divided according to weight change between baseline and follow-up, which produced groups experiencing weight loss (loss of ≥5% of initial body weight), weight gain (gain of ≥5% of initial body weight), and relatively little weight change (weight maintained within 5% of initial body weight). Characteristics of the three groups were compared using one-way analyses of variance (ANOVAs) for continuous variables and chi-square tests for categorical variables. Logistic regression analyses were used to calculate the odds of depressed mood, low wellbeing, hypertension, and high serum triglycerides at follow-up, with the weight stable group as the reference group. All models were adjusted for baseline weight, baseline status on the outcome variable, age, sex, wealth, and weight loss intention. Ethnicity was not included as a covariate because 98% of participants were white British; we ran analyses adjusting for ethnicity, which did not affect the results.

Because there is not an established cut-off for dichotomising wellbeing scores, data were also analysed continuously for this outcome using repeated-measures ANOVA (controlling for baseline weight, age, sex, wealth, and weight loss intention) to check whether the logistic regression results were affected by the reduced power that comes with dichotomizing data.

Two additional sets of logistic regression models were used to control for possible confounding by ill health and life stresses over the weight change period. In addition to the covariates entered into the earlier models, the first set of models adjusted for changes in health status, including reporting a limiting long-standing illness at follow-up (those with limiting long-standing illness at baseline had already been excluded), developing stroke, diabetes, chronic lung disease, cancer, or coronary heart disease between baseline and follow-up, and dying within 21 months of follow-up. The second set of models adjusted for three major life events: marital breakdown, being widowed, and experiencing the death of a parent between baseline and follow-up.

To check that any observed differences in outcomes between the groups were not simply reflective of underlying differences between the groups, we repeated the analyses using inverse probability of treatment weighting (IPTW). Estimated inverse-probability weights were calculated to reflect the probability of a participant losing weight, remaining weight stable, or gaining weight given all included covariates. Multinomial logistic regression models used these inverse-probability weights to estimate the effect of weight loss on depressed mood, wellbeing, hypertension, and triglycerides, achieving balance across weight change groups on all covariates. No differences in results were observed (data not shown).

All statistical analyses were done using IBM SPSS Statistics version 19, with the exception of the IPTW models which were done in STATA version 13. We considered two-sided *p* values <.05 to be statistically significant.

## Results

### Sample characteristics

Of the 1979 overweight and obese participants, 278 (14%) lost at least 5% of their initial body weight between baseline and follow-up, 1408 (71%) were weight stable within 5%, and 293 (15%) gained at least 5% of their initial weight. Mean weight change was −6.8 kg (SD 3.0) in the weight loss group (−8% of initial body weight, range =  −22.4 kg to −3.3 kg), +0.2 kg (SD 2.1) in the weight stable group (+0% of initial body weight, range =  −5.8 kg to +5.2 kg), and +6.4 kg (SD 2.5) in the weight gain group (+8% of initial body weight, range = +3.3 kg to +15.9 kg). There was no sex difference in mean percentage weight change (*p* = .447).

Demographic and anthropometric characteristics of the three weight change groups are summarized in Table 1. Mean age was highest in the weight loss group and lowest in the weight gain group (*p*<.001). There were higher proportions of men (*p*<.001) and more wealthy participants (*p* = .012) in the weight stable group than in the other two groups. The weight stable group was significantly taller than either of the other groups (*p*<.001), and the weight gain group had the lowest mean weight at baseline (*p* = .015). The weight loss group had the highest mean BMI (*p* = .021) at baseline, and the highest proportion of obese participants (*p* = .022). The groups did not differ in the proportion who had reported an intention to lose weight in 1998 (*p* = .743).

**Table 1:** Demographic and anthropometric characteristics at baseline by weight change tertile – mean (SD), percentage (*n*).

	Weight loss	Weight stable	Weight gain	
	(*n* = 278)	(*n* = 1408)	(*n* = 293)	*p*
**Demographic characteristics**				
Age (years)	65.82 (8.93)	63.91 (7.87)	61.70 (7.02)	<.001
Sex				
Male	45.3% (126)	54.0% (761)	41.0% (120)	<.001
Female	54.6% (152)	46.0% (647)	59.0% (173)	-
Wealth quintiles				
1 (lowest)	16.5% (46)	9.7% (137)	13.0% (38)	.011
2	14.0% (39)	14.8% (209)	19.5% (57)	-
3	23.7% (66)	21.6% (304)	21.5% (63)	-
4	21.6% (60)	25.1% (354)	24.6% (72)	-
5 (highest)	23.4% (65)	26.5% (373)	20.5% (60)	-
**Anthropometric characteristics**				
Height (cm)	164.98 (9.72)	167.19 (9.61)	165.26 (8.93)	<.001
Weight (kg)	81.26 (13.42)	81.79 (12.57)	79.45 (11.75)	.015
BMI (kg/m^2^)	29.78 (3.76)	29.21 (3.43)	29.04 (3.31)	.021
Weight status				
Overweight	60.1% (167)	67.5% (950)	70.3% (206)	.022
Obese	39.9% (111)	32.5% (458)	29.7% (87)	-
Weight loss intention				
Trying to lose weight	53.2% (148)	52.4% (738)	53.9% (158)	.743
Not trying to lose weight	46.4% (129)	47.1% (663)	46.1% (135)	-

Note: Numbers may not sum to the total group number, as some items were not answered by all participants. Percentages were derived from the full group and may therefore not sum to 100%.

There were no significant group differences at baseline in the proportion who had depressed mood (*p* = .540). However, the weight gain group had lower baseline prevalence of hypertension (*p* = .008) and high triglycerides (*p* = .035), and a higher baseline rate of low wellbeing (*p* = .005) than the weight loss and weight stable groups.

### Psychological wellbeing at follow-up

**Figure 1:**
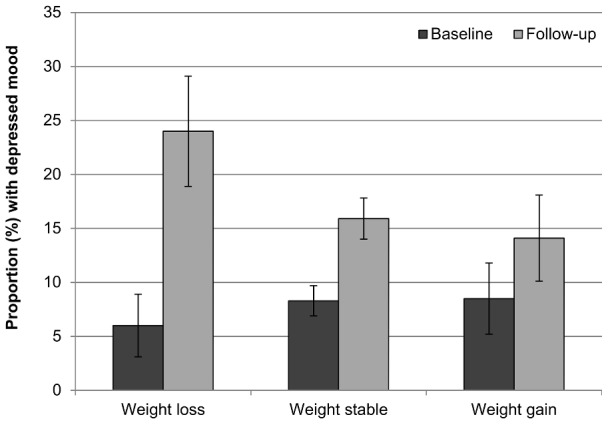
Prevalence of depressed mood at baseline and follow-up by weight change status. Values are mutually adjusted for age, sex, wealth, and weight loss intention. Error bars represent the 95% confidence interval.

**Figure 2:**
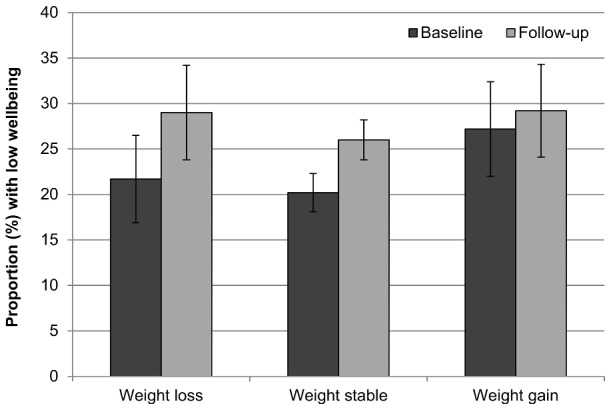
Prevalence of low wellbeing at baseline and follow-up by weight change status. Values are mutually adjusted for age, sex, wealth, and weight loss intention. Error bars represent the 95% confidence interval.

Figure 1 shows the proportion of participants with depressed mood in each group at each time point, and Figure 2 shows the proportion with low wellbeing. Psychological wellbeing deteriorated (increased rates of depressed mood and low wellbeing) between baseline and follow-up across all three weight change groups. However, the proportion with depressed mood increased more in the weight loss group than in those whose weight was stable or who gained weight (+289%, +86%, and +62% respectively). Compared with participants who were weight stable, the odds of depressed mood at follow-up (controlling for baseline weight, baseline depressed mood, age, sex, wealth, and weight loss intention) were almost 80% higher for weight losers (OR = 1.78, 95% CI = 1.29–2.47, *p* = .001), but were not significantly different for weight gainers (OR = 0.86, 95% CI = 0.59–1.26, *p* = .441).

Rates of low wellbeing also appeared to increase more in the weight loss group than in the other two groups (+31%, +22%, −4%), but odds of low wellbeing at follow-up compared with people who were weight stable were not significantly higher for weight losers (OR = 1.16, 95% CI = 0.81–1.66, *p* = .430), nor were they significantly different for those who gained weight (OR = 0.98, 95% CI = 0.69–1.39, *p* = .889).

**Figure 3:**
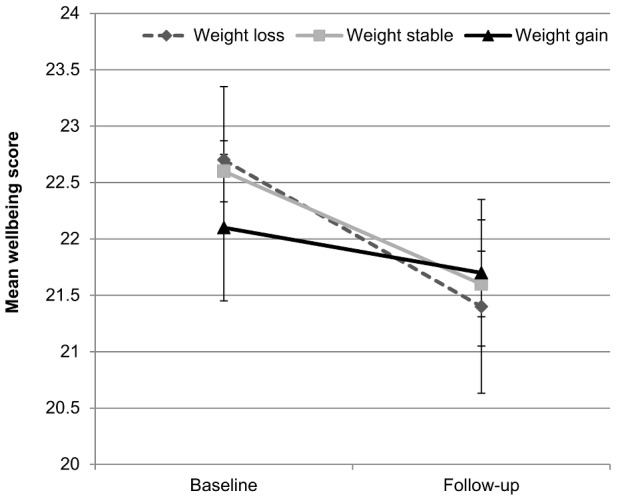
Mean wellbeing at baseline and follow-up by weight change status. Values are mutually adjusted for age, sex, wealth, and weight loss intention. Error bars represent the 95% confidence interval.

However, when wellbeing data were analysed continuously, there was a borderline-significant interaction between weight change group and time (*F*(2, 1685) = 2.72, *p* = .066), indicating that the decline in wellbeing over time may differ by group. Figure 3 shows that mean wellbeing declined most between baseline and follow-up in the weight loss group, declined less in the weight stable group, and showed little change in the weight gain group.

### Cardio-metabolic risk at follow-up

**Figure 4:**
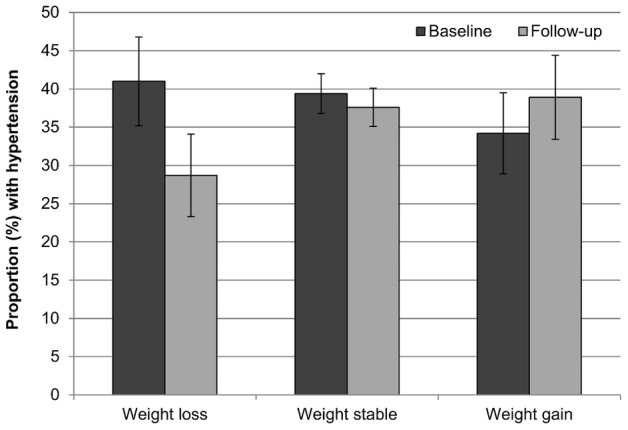
Prevalence of hypertension at baseline and follow-up by weight change status. Values are mutually adjusted for age, sex, wealth, and weight loss intention. Error bars represent the 95% confidence interval.

**Figure 5:**
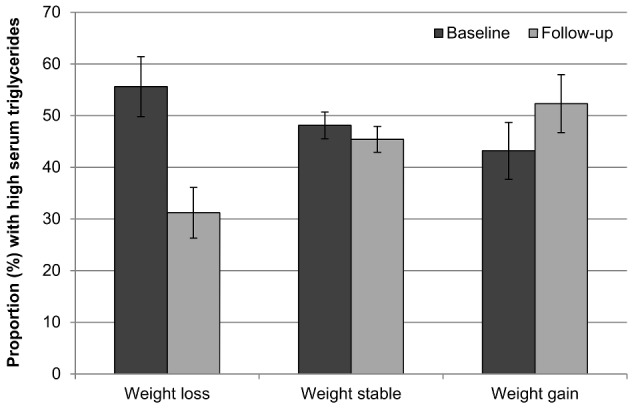
Prevalence of high serum triglycerides at baseline and follow-up by weight change status. Values are mutually adjusted for age, sex, wealth, and weight loss intention. Error bars represent the 95% confidence interval.

Figure 4 and Figure 5 show the proportion of participants with hypertension and high serum triglycerides respectively in each group at each time point. Consistent with trial data, weight loss was associated with improvement in both indicators of cardio-metabolic risk. The proportion of participants with hypertension decreased in the weight loss group, changed very little in the weight stable group, and increased in the weight gain group (−28%, −4%, +18%). The proportion with raised serum triglycerides also decreased in the weight loss group and increased in the weight gain group (−47%, −13%, +5%). Compared with those who were weight stable, the odds of being hypertensive at follow-up were nearly 40% lower for those who lost weight (OR = 0.61, 95% CI = 0.45–0.83, *p* = .002), but not significantly higher for those who gained weight (OR = 1.17, 95% CI = 0.88–1.56, *p* = .284). Similarly, the odds of raised triglycerides relative to the weight stable group were around 60% lower in weight losers (OR = 0.41, 95% CI = 0.28–0.60, *p*<.001), and were almost 60% higher in weight gainers (OR = 1.57, 95% CI = 1.11–2.22, *p* = .011).

### Confounding by changes in health status and major life events

Analyses were repeated adjusting for changes in health status and major life events in order to test for confounding of the associations between weight loss and poorer psychological outcomes.

Between baseline and follow-up, 106 participants (5%) developed diabetes, 81 (4%) developed coronary heart disease, 67 (3%) developed cancer, 45 (2%) developed chronic lung disease, and 43 (2%) had a stroke. An additional 257 participants (13% of the sample) who had not developed any of these health conditions reported a limiting long-standing illness at follow-up, and 35 participants (2%) died within 21 months of follow-up. In models that controlled for these changes in health status, associations between weight loss and cardio-metabolic outcomes were virtually unchanged from previous analyses, with reduced odds of hypertension (OR = 0.60, 95% CI = 0.44–0.83, *p* = .002) and high triglycerides (OR = 0.39, 95% CI = 0.26–0.58, *p*<.001). Similarly, there was still a significant association between weight loss and increased odds of depressed mood (OR = 1.52, 95% CI = 1.07–2.17, *p* = .019), although the odds ratio was lower than when changes in health status were not controlled for (OR = 1.78). Post-hoc analyses indicated that this difference was largely accounted for by the inclusion of limiting long-standing illness, diabetes, and cancer in the model (OR excluding these variables = 1.71). Consistent with earlier analyses, there was no significant association between weight loss and wellbeing in the logistic regression model (OR = 1.11, 95% CI = 0.77–1.61, *p* = .577), but the repeated-measures ANOVA run on continuous wellbeing scores revealed a significant group-by-time interaction (*F*(2, 1628) = 3.06, *p* = .047), indicating a greater decline in wellbeing in the weight loss group. These results suggest that associations between weight loss and increased rates of depressed mood and decreased wellbeing scores are not fully explained by changes in health status.

Between baseline and follow-up, 9 participants (1%) were legally separated or divorced, 41 (2%) were widowed, and 100 (5%) experienced the death of one or both parents. Controlling for potential confounding by these major life events had little effect on associations between weight loss and any of the outcome variables. Significant associations remained with hypertension (OR = 0.55, 95% CI = 0.40–0.76, *p*<.001), high triglycerides (OR = 0.38, 95% CI = 0.25–0.57, *p*<.001), and depressed mood (OR = 1.83, 95% CI = 1.29–2.58, *p* = .001). There was no significant association between weight loss and wellbeing in the logistic regression model (OR = 1.13, 95% CI = 0.77–1.66, *p* = .536) but the group-by-time interaction was significant for changes in wellbeing scores (*F*(2, 1463) = 3.32, *p* = .036), with a greater reduction in wellbeing over time in the weight loss group.

## Discussion

This study is the first to examine associations between weight loss and change in both psychological wellbeing and cardio-metabolic risk in overweight and obese older adults from a nationally-representative, population-based cohort. Around 15% of these overweight and obese adults lost ≥5% of their bodyweight and a similar proportion gained ≥5%. The group who lost at least 5% of their initial body weight derived the well-established cardio-metabolic benefits of weight loss, with rates of hypertension and high serum triglycerides declining [8], [9]. However, there was no evidence that weight loss was associated with improved psychological wellbeing. In fact, significantly more of the weight loss group than the groups who were weight stable or gained weight had depressed mood at follow-up, and at least in some of the adjusted analyses, more had low wellbeing.

Our finding that weight loss was not associated with favorable psychological effects is similar to results from two other population-based studies, the Health and Retirement Study and the Health ABC study; neither of which found that weight loss was associated with improved mood, and which found some evidence that it was associated with increased depressive symptoms [13], [14]. However, both of these studies included participants across the full weight spectrum. We had expected that by restricting our a sample to participants who were overweight or obese at baseline, evidence of adverse psychological effects of weight loss would disappear. However, the present findings indicate that even among overweight and obese older adults, a group for whom weight loss is recommended, there is no evidence for positive effects of weight loss on mood.

There are three potential explanations for the association between weight loss and depressed mood: 1) weight loss causes depressed mood; 2) depressed mood causes weight loss; 3) weight loss and depressed mood share a common cause. If we take the first explanation to be correct, there is a clear contrast between the present finding of an adverse association between weight loss and mood, and trial data that typically show at least modest improvements in depression during clinical weight loss interventions [11], [12] (although few trials have a comparable length of follow-up for psychological effects). One possible explanation for the difference is that the mood improvements in clinical trials are a consequence of the supportive treatment context rather than weight loss *per se.* This is consistent with the observation that mood improvements often occur early in treatment, prior to achieving significant weight loss [32], and that mood improvement is independent of the amount of weight lost [11], [33]. Depression rates also tend to be higher in obese individuals seeking treatment than in community obese samples, giving more scope for improvement [34].

Are there possible mechanisms for adverse psychological effects of weight loss? The poor long-term maintenance of weight loss is notorious [35], and in itself could be interpreted as demonstrating that the personal costs of losing weight exceed the benefits. Resisting food in environments that offer abundant eating opportunities requires sustained self-control, and given that self-control appears to be a limited resource [36], other areas of life may suffer as a consequence. Loss of fat stores may also initiate signals for replenishment of adipocytes [37], thereby stimulating hunger and appetite and making weight control progressively more difficult [38]. These observations suggest that weight loss is a significant psychobiological challenge, and as such, could affect psychological wellbeing.

Because our results are correlational, it is equally possible that the second explanation is correct, and weight loss was driven by a decline in mood. Evidence from the clinical literature is suggestive of a causal relationship in this direction, with major depressive disorder often associated with significant weight loss [39], and treatment with antidepressant medication leading to weight gain [40]. Population studies have also demonstrated longitudinal associations between depressive symptoms and weight loss [13], [41]. Depressed mood may cause weight loss directly or indirectly through changes in appetite [39] or level of physical activity [42].

From the present analyses, it is not possible to draw firm conclusions on the direction of causation. Longitudinal data can offer insight on causality, as they allow examination of the temporality of an association (i.e. whether A precedes B, or B precedes A). However, because our analysis was of change in weight loss and change in mood over the same times (with variables derived from data at both time points), results are only correlational and causal interpretations are limited. To determine the direction of causation, at least three time points are needed, making it possible to examine whether weight loss precedes the onset of depressed mood, or vice versa.

The third possibility is that the association between weight loss and decline in mood was not causal, but was due to them sharing a common (separate) cause. We investigated some potential common causes in terms of health and life stress. We adjusted for changes in health status and major life events occurring between baseline and follow-up. Although this slightly reduced the magnitude of the association between weight loss and depressed mood, it remained significant, and there was little change in the association between weight loss and wellbeing; suggesting that changes in the health and life stress variables we controlled for did not explain the association. However, it is important to acknowledge that other health issues or life events than the ones assessed, or potential underlying causes other than health issues or life events, may be a common factor causing both weight loss and depressed mood, so we are not able to conclusively rule out this explanation. The limitations of the ‘common cause’ measures are discussed in more detail below.

### Limitations

Our study had several important limitations. The information on intention to lose weight had been collected six years before the wave used as the baseline in these analyses, as part of the 1998 HSE that one third of the ELSA sample were recruited from. As such, it is likely that some participants who had been intending to lose weight in the past had given up trying and others who had not been intending to lose had started doing so. However, these results are consistent with findings from the Health ABC study, which also found that controlling for a more proximal measure of intentionality did not affect the finding of adverse psychological effects of weight loss [14]. We also found no evidence that the psychological effects of weight loss were more positive for people who were obese (rather than merely overweight) at baseline, yet the former would certainly be more likely to be intending to lose weight [43]. The use of an arbitrary, unvalidated threshold to indicate low levels of wellbeing is also problematic, and we observed some differences in results when wellbeing data were analysed continuously, so these results should be interpreted with caution. In addition, although we used an established, widely-used threshold to define depressed mood [18], it is important to note that opinions on what constitutes a high level of depressive symptomatology may differ.

Like all cohort studies, there may be confounding by unmeasured or imprecisely measured factors. A potential confounder in the present analyses is the presence of underlying disease causing both weight loss and depressed mood. Naturally, we were not able to exclude individuals with undiagnosed disease, but we excluded those with a diagnosis of a limiting long-standing illness at baseline, and repeated the analyses controlling for changes in health status between baseline and follow-up, and death in the 21 months after follow-up, without this changing the conclusions substantially. This suggests that this effect was not solely due to ill health driving changes in weight and changes in mood. However, it is important to note that limiting long-standing illness was self-reported and therefore only offers a crude indication of health status.

It was also possible that associations between weight loss and depressed mood were due to major life events which caused both depressed mood and weight loss. Getting divorced, being widowed, or experiencing the loss of a loved one are major events. However, very few participants in the sample experienced an event of this type over the period under investigation and adjusting for their influence had little effect on the results, although data on bereavement were limited to parents and spouses. It would have been useful to have been able to widen the analyses to control for death of children or close friends, but this information was not available. Adjusting for these major life events was problematic in that they could not have been experienced by participants who were not married at baseline or whose parents died before baseline. Another important life event that might cause both a decline in mood and weight loss is job loss, but this is difficult to quantify in this age group because the same outcome (i.e. no longer working) might be seen as a positive change for some individuals (e.g. voluntary retirement) and a negative change for others (e.g. redundancy). There are also multiple more idiosyncratic stressors which were not assessed.

The ELSA sample comprises predominantly white, older adults and the effects of weight loss may be different among younger adults or different ethnic groups. In addition, the present analyses were necessarily restricted to participants who had data available at both time points, and in line with retention in other longitudinal studies, the analysed sample was slightly younger, wealthier, and healthier than the total ELSA sample [44]. As a consequence of this healthy participant bias, results may not be population-representative and caution should be taken in extrapolating to other populations.

### Conclusions

The results of this study indicate that overweight or obese adults who experience a 5% reduction in body weight over a four-year period obtain no psychological benefit and may be at risk of increased depression, despite benefiting from the expected reductions in cardio-metabolic risk. This finding has implications for clinical practice and indicates that health professionals should take note of their patients’ psychological wellbeing alongside their physical health when recommending or responding to weight loss.
